# Association between breakfast patterns and executive function among adolescents in Shanghai, China

**DOI:** 10.3389/fnut.2024.1373129

**Published:** 2024-05-14

**Authors:** Xuelai Wang, Shuangxiao Qu, Dongling Yang, Wenjuan Qi, Fengyun Zhang, Rong Zhu, Lijing Sun, Qiong Yan, Yue Qi, Guizhen Yue, Cancan Yin, Chunyan Luo

**Affiliations:** Division of Child and Adolescent Health, Shanghai Municipal Center for Disease Control and Prevention, Shanghai, China

**Keywords:** breakfast patterns, skipping breakfast, adolescents, executive function, latent class analysis

## Abstract

**Introduction:**

The aim of this cross-sectional study was to investigate the association between breakfast patterns and executive function among adolescents in Shanghai, China.

**Methods:**

In 2022, we randomly recruited 3,012 adolescents aged 12–13 years from all administrative districts in Shanghai. Breakfast information was collected by parents using a one-day recall method. Executive function was measured using the Behavior Rating Inventory of Executive Function-Parent Version. Latent Class Analysis was performed to identify breakfast patterns based on the food groups in the Diet Quality Questionnaire for China.

**Results:**

Breakfast patterns were classified into three categories: “Egg and milk foods”, “Grain foods”, and “Abundant foods”, except for adolescents who skipped breakfast. Logistic regression was used to estimate the multivariate odds ratio (ORs) and 95% confidence intervals (95% CI) for the association between breakfast patterns and potential executive dysfunction. Adolescents in the “Abundant foods” class had a lower risk of executive dysfunction in terms of initiate (OR: 0.36; 95% CI: 0.17–0.76), and organization of materials (OR: 0.18; 95% CI: 0.04–0.94), compared to those who skipped breakfast. Similarly, the breakfast patterns of “Grain foods” and “Egg and milk foods” were associated with a lower risk of executive dysfunction, including initiate and working memory.

**Discussion:**

Our findings suggest that breakfast patterns were associated with executive function. The improvement of breakfast patterns among adolescents should be a significant public health intervention.

## 1 Introduction

Adolescence is a transitional period between childhood and adulthood. Health during this period has an impact throughout the life course (1). The adaptive plasticity of adolescence offers an opportunity to rectify problems that have arisen from earlier life experiences (2). On the other hand, health-related behaviors typically begin or are reinforced during adolescence. These behaviors can have long-term effects on adolescents’ health as adults and increase the burden of disease in adulthood (3). However, adolescent health does not receives adequate attention because adolescence is often considered the healthiest time of life (4). Therefore, more research and evidence-based interventions are needed to improve the health of adolescents.

Adolescence is an important developmental period for maturation in brain function, particularly cognitive abilities (5). Executive function, also called cognitive control, refers to a set of cognitive processes that allow individuals to plan, monitor, and achieve short- and long-term goals (6). These executive processes are essential for almost every aspect of daily life. More importantly, numerous studies have found a longitudinal relationship between executive function with pronounced social skills and academic achievement (7, 8). Executive function skills emerge during the first few years of life and strengthen significantly throughout childhood, adolescence, and into early adulthood (9). The development of executive function is influenced by a variety of biological and environmental factors (10). Nutrition is a readily modifiable factor that can impact brain maturation in school-aged children (11). Thus, effective strategies are needed to enhance the development of executive function skills.

Breakfast is often considered the most important meal of the day. Dietary guidelines generally state that breakfast provides 20–25% of daily energy intake (12). Increasing evidence suggests that consuming breakfast has a positive impact on school performance and cognitive function (12, 13). Particularly, high-quality breakfast patterns were found to be associated with better cognitive performance among adolescents (14–16). However, breakfast is the most frequently skipped meal among adolescents worldwide (17). An Australian census data showed that more than 27% of grade 4–12 students reported often skipping or always skipping breakfast (18). Furthermore, the nutritional quality of breakfast was relatively poor among adolescents. A survey of six major cities in China indicated that only 41.7% of students consumed a nutritious breakfast (19). It can be seen that adolescents and the general public lack awareness of the importance of breakfast.

In order to improve the situation of breakfast among adolescents in China, the latest Dietary Guidelines for Chinese School-age Children recommend that school-age children should have breakfast daily, and consume a nutritious breakfast with a variety of foods (20). It also emphasizes that a nutritious breakfast can improve cognitive performance. In fact, the Chinese dietary pattern has shifted significantly in recent decades (21). On the basis of traditional carbohydrate-rich foods, breakfast patterns have transitioned to a modern diet with a high intake of eggs and dairy products (22). To our knowledge, few studies have identified and described breakfast food consumption patterns and examined their association with executive function among adolescents in China. Therefore, in the current study, we described the most frequently observed breakfast patterns among adolescents in Shanghai, China. Furthermore, we aimed to test the hypothesis that better breakfast patterns, including breakfast foods, are associated with good executive function performance, by a cross-sectional study among adolescents aged 12 to 13 years.

## 2 Materials and methods

### 2.1 Study design and population

Data was obtained from the 2022 Surveillance of Common Diseases and Health Influencing Factors (SCDHIF) of Students in Shanghai, China, which was an annual cross-sectional survey conducted by the Shanghai Municipal Center for Disease Control and Prevention (SCDC) (23). The 2022 SCDHIF was conducted from October 2022 to March 2023. The survey samples covered all administrative districts in Shanghai, including seven urban areas (districts of Huangpu, Xuhui, Jing’an, Changning, Hongkou, Yangpu, and Putuo) and nine suburbs (districts of Jinshan, Pudong, Fengxian, Minhang, Songjiang, Jiading, Qingpu, Chongming, and Baoshan).

We recruited participants aged 12 to 13 years old using a multi-stage cluster sampling. The sample size was calculated as follows:


$$n=\frac{p\left(1-p\right)Z_{\alpha /2}^{2}}{d^{2}}=1411$$

where *p* is the percentage of middle school students who do not have breakfast every day in Shanghai (2021), with 21.4%. The *Z* critical value for a 95% confidence interval is 1.96 for a two-tailed test. *d* is margin of error, which was cited as 0.1*p. The sample size was doubled to 2,822 in 16 districts, considering the gender stratification. The minimum sample size for each district was 177 participants, so we randomly selected one junior high school from each district. Due to an insufficient number of students sampled from schools in the Huangpu and Jinshan districts, we randomly selected an additional school from these districts. We then randomly sampled entire classes from the 7th grade of the selected schools to meet the minimum sample size requirement. As this survey was conducted during the COVID-19 pandemic period. We were allowed to enter the school only when there were no students or teachers infected with COVID-19. If a COVID-19 case was found in school, students were required to undergo home quarantine. The survey was postponed until students were allowed to return to school. Therefore, the participants in this study were either not infected with COVID-19 or had recovered from COVID-19. Finally, a total of 3,012 students participated in the survey.

**Figure 1:**
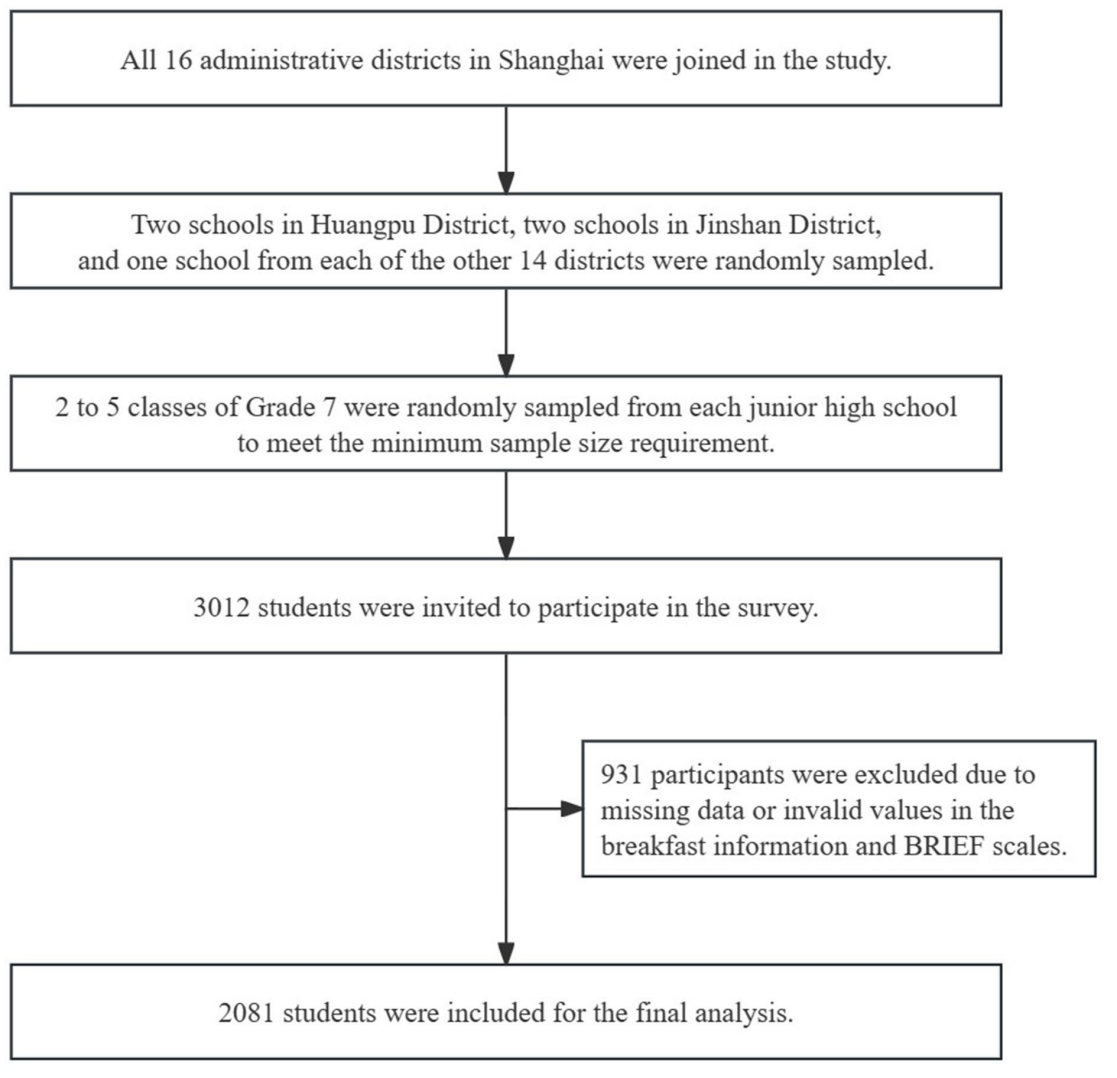
Flowchart of participant inclusion.

At the beginning of the survey, SCDC organized a training for investigators from district-level CDCs. Then, the investigators in each district randomly sampled one or two schools and obtained written informed consent from the school principal. The health teacher and class teachers assisted in the implementation. Participants’ parents were asked to record breakfast foods and complete the Behavioral Rating Inventory of Executive Function (BRIEF) questionnaire. All participants were assured of the anonymity and confidentiality of the information provided in the survey, and they were free to discontinue their participation at any time during the study. Participants who had completed the questionnaire and valid BRIEF scales were eligible. Those with incomplete questionnaire data or invalid BRIEF scales were excluded (Figure 1).

### 2.2 Breakfast information collection

**Figure 2:**
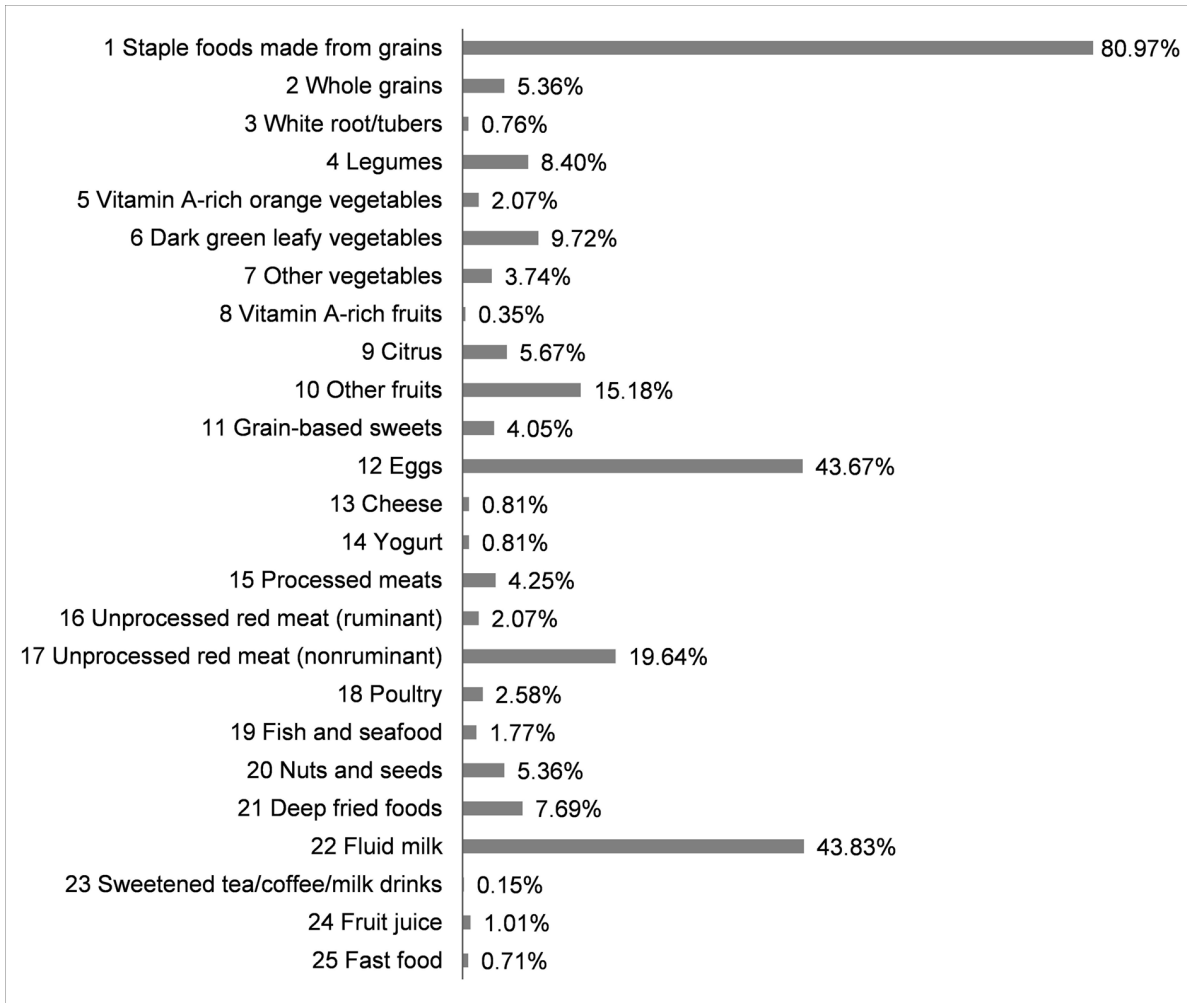
Percentage of DQQ food groups consumed by participants who had breakfast (%).

Breakfast information was recorded for one day by parents. Parents were asked to record breakfast information for one day by scanning a QR code and entering it into an online platform. The parents or caregivers who could not access the online platform used paper forms instead. According to the breakfast recording, we initially divided participants into eaten group and skipped group. For the eaten group, breakfast consumption was coded into 29 food groups using the Diet Quality Questionnaire (DQQ) tool, which has been adapted to represent foods in the Chinese context (24). DQQ for China contains commonly-consumed foods as follows: 01, staple foods made from grains; 02, whole grains; 03, white root/tubers; 04, legumes; 05, vitamin A-rich orange vegetables; 06, dark green leafy vegetables; 07, other vegetables; 08, vitamin A-rich fruits; 09, citrus; 10, other fruits; 11, grain-based sweets; 12, other sweets; 13, eggs; 14, cheese; 15, yogurt; 16, processed meats; 17, unprocessed red meat (ruminant); 18, unprocessed red meat (nonruminant); 19, poultry; 20, fish and seafood; 21, nuts and seeds; 22, packaged ultra-processed salty snacks; 23, instant noodles; 24, deep fried foods; 25, fluid milk; 26, sweetened tea/coffee/milk drinks; 27, fruit juice; 28, sugar-sweetened beverages (SSBs) (sodas); 29, fast food. The final breakfast consumption for participants in the eaten group comprised twenty-five food groups (Figure 2).

### 2.3 Assessment of executive function

In the current study, we used Behavioral Rating Inventory of Executive Function (BRIEF) Parent Form to evaluate adolescents’ executive function in the past 6 months. This questionnaire offers an ecological assessment of executive function behaviors in the home environments of children aged 5 to 18 years, which is widely used in epidemiological studies (25, 26). The Chinese version of BRIEF demonstrates high internal consistency, with a reliable test–retest of 0.68–0.89, internal consistency of 0.74–0.96, and internal consistency of 0.74–0.96 (27). The BRIEF consists of 86 items grouped into eight clinical domains of executive function, including inhibit, shift, emotional control, initiate, working memory, plan/organize, organization of materials, and monitor. These clinical scales combine to form two indexes: the Behavioral Regulation Index (BRI) and the Metacognition Index (MI), as well as one composite summary score, the Global Executive Composite (GEC). The BRI consists of the Inhibit, Shift, and Emotional Control scales, while the MI consists of other scales. The GEC is a summary score of all eight clinical scales. According to the BRIEF professional manual, all clinical scales and indexes were converted into *t*-scores adjusted for age and sex. A higher *t*-score indicates more executive function problems. Scores ≥60 (e.g., 1 SD from the mean) were classified as “elevated executive dysfunction” (25).

### 2.4 Covariates

We collected data on participants’ gender, maternal education, family affluence status, participants’ sleep duration, and whether it was a school day or weekend. Maternal education was categorized as senior high school or below, college, undergraduate, and graduate. Information on family socioeconomic status (SES) was collected separately using the Family Affluence Scale (FAS), which has been proven to be a reliable and valid measure of SES for adolescents in China (28). The FAS scale was categorized into three levels of affluence: low, middle, and high. Sleep duration was found to be associated with breakfast patterns among adolescents (29). Thus, we collected bedtime from the night before and wake-up time from the day to calculate sleep duration. The National Sleep Foundation recommends that children aged 6–13 years should get between 9 and 11 h of sleep (30). The sleep duration in the current study was categorized as “<9 h” and “≥9 h”. In addition, we distinguished between school-day and weekends based on the filling date, as different schedules may affect wake-up time and breakfast time.

### 2.5 Statistical analyses

We conducted Latent Class Analysis (LCA) to identify mutually exclusive classes of breakfast patterns based on the consumption of 25 food groups using the software Mplus (version 8.3). This approach enables the identification of distinct profiles adopted by subgroups of individuals who follow similar breakfast patterns. The classes can then be used to determine whether a specific breakfast pattern is associated with elevated executive dysfunction outcomes. The best fitting latent class was selected based on the model Akaike Information Criteria (AIC), Bayesian Information Criteria (BIC), Adjusted Bayesian Information Criteria (aBIC), entropy, Lo–Mendell–Rubin (LMR) and Bootstrapped Likelihood Ratio Tests (BLRT). Lower values of AIC, BIC, and aBIC, along with higher entropy, indicate a better model fit. LMR and BLRT compare *k* and *k* − 1 class models. A low and significant *p* value indicates that the *k* model is superior to the *k* − 1 class model (*p* < 0.05).

Data were presented as the mean and standard deviation (SD) for continuous variables, or as the number and percentage for categorical variables. To examine the differences in characteristics of breakfast patterns, a Chi-square test for categorical variables was conducted. We used binary logistic regression to examine the association of breakfast patterns with executive function, with breakfast skipping as the reference group. The unadjusted model was first used. We further adjusted for maternal education (high school or less, junior college, undergraduate, graduate, and missing value), family affluence status (low, middle, high, and missing value), sleep duration (<9 h and ≥9 h), and school day (yes and no). The results of the logistic regression analysis were presented as odds ratio (OR) and 95% confidence interval (95% CI).

Additionally, to assess potential effect modification, we performed stratified analyses by gender (boys, girls), maternal education (below undergraduate, undergraduate), and family affluence status (low, middle, high). We tested the statistical significance of the interactions using the likelihood ratio test. We also conducted a sensitivity analysis to test the robustness of our findings by excluding individuals with missing values. Data analysis was conducted using Stata version 14 software (StataCorp, College Station, TX), and a two-sided *p*-value of <0.05 indicated statistical significance.

## 3 Results

### 3.1 LCA of breakfast food groups

Breakfast patterns were identified using LCA. A 5-class model was initially tested, but it did not yield the optimal LMR-LRT and BLRT values, so it was not considered further. Both the 3-class and 4-class models showed significant *p*-values for LMR-LRT and BLRT tests. However, the 3-class model presented lower AIC, BIC, and aBIC values, suggesting a better fit for the current study (Table 1).

**Table 1:** Model-fit indexes for latent class analysis models.

LCA model	*df*	AIC	BIC	aBIC	Entropy	LMR	BLRT	Classification probability
2	51	21297.354	21582.359	21420.330	0.843	<0.001	<0.001	0.198/0.802
3	77	21005.208	21435.508	21190.876	0.795	<0.001	<0.001	0.089/0.179/0.732
4	103	20958.476	21534.073	21206.838	0.698	0.009	<0.001	0.083/0.178/0.088/0.652
5	129	20893.632	21614.526	21204.687	0.739	0.480	<0.001	0.070/0.116/0.645/0.073/0.096

df, degrees of freedom; AIC, Akaike information criteria; BIC, Bayesian information criteria; aBIC, adjusted Bayesian information criteria; LMR, Lo–Mendell–Rubin; BLRT, bootstrapped likelihood ratio tests.

**Figure 3:**
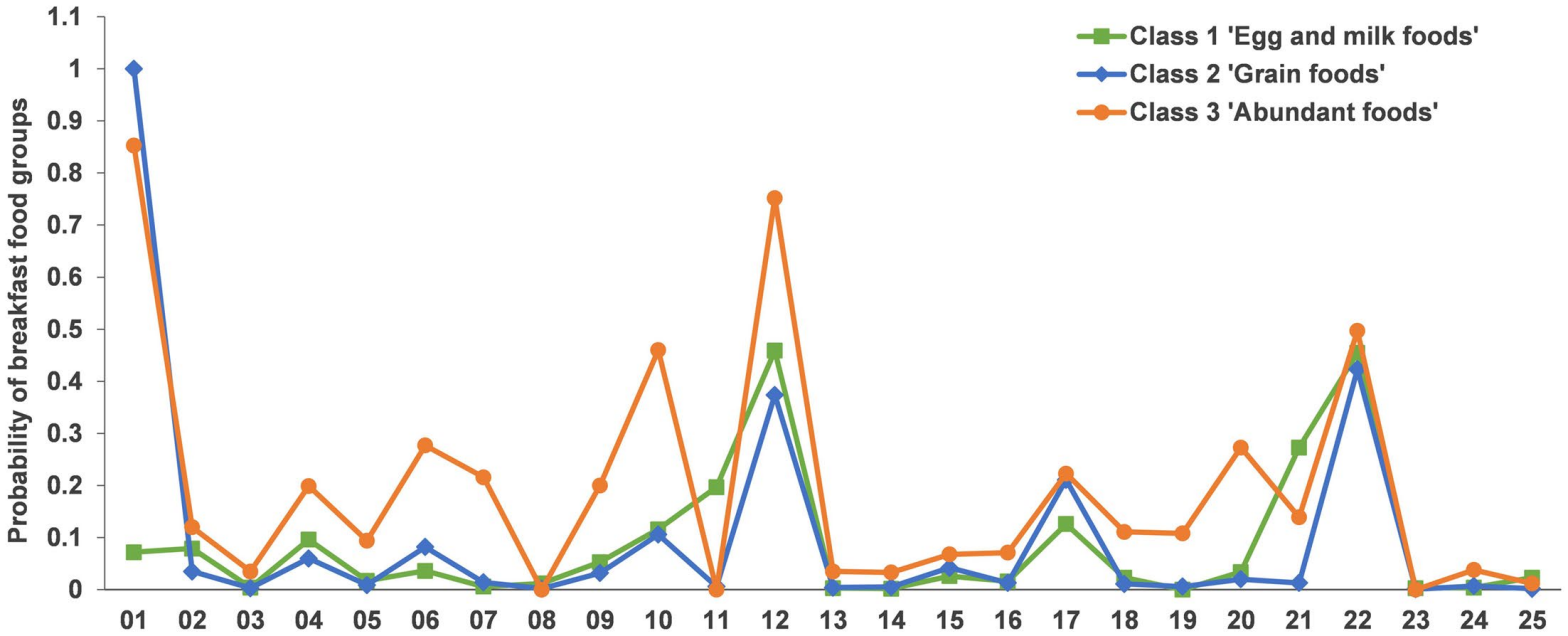
Response probabilities of breakfast intake food groups in three classes. 01, Staple foods made from grains; 02, Whole grains; 03, White root/tubers; 04, Legumes; 05, Vitamin A-rich orange vegetables; 06, Dark green leafy vegetables; 07, Other vegetables; 08, Vitamin A-rich fruits; 09, Citrus; 10, other fruits; 11, grain-based sweets; 12, eggs; 13, cheese; 14, yogurt; 15, processed meats; 16, unprocessed red meat (ruminant); 17, unprocessed red meat (nonruminant); 18, poultry; 19, fish and seafood; 20, nuts and seeds; 21, deep fried foods; 22, fluid milk; 23, sweetened tea/coffee/milk drinks; 24, fruit juice; 25, fast food.

Class 1 (*n* = 354, 17.92% of breakfast eaten group) was characterized by a medium level of eggs and fluid milk, and a low level of other food groups. This class was designated as the “Egg and milk foods” class. The largest class, Class 2 (*n* = 1,445, 73.16% of breakfast eaten group) was characterized by the highest consumption of staple foods made from grains, such as rice, noodles, steamed buns, and bread. We designated it as the “Grain foods” class. Class 3 (*n* = 176, 8.91% of breakfast eaten group) was characterized by the highest levels of eggs, fluid milk, vegetables, fruits, meats, seafood, nuts, and seeds, as well as a high level of staple foods made from grains. Class 1 was designated as the “Abundant foods” class (Figure 3).

### 3.2 Characteristics of study participants

Characteristics of study participants across categories of breakfast intake are shown in Table 2. Boys were more likely to be classified under the “Egg and milk foods” class, while girls were more inclined to be categorized under the “Grain foods” and “Abundant foods” classes. Participants who consumed breakfast were more likely to come from middle-class socioeconomic families, have better maternal education, and have shorter sleep duration (Table 2). The description of BRIEF scores of participants belonging to different breakfast patterns is presented in Supplementary Table S1.

**Table 2:** Study population characteristics according to breakfast patterns.

Characteristics	Total	Skipping breakfast	Latent classes of breakfast consumption*			*p*
			Class 1	Class 2	Class 3	
*N*	2,081	106	354	1,445	176	
Gender (%)						
Boys	49.18	59.43	54.52	47.20	48.59	0.013
Girls	50.82	40.57	45.48	52.80	51.41	
Maternal education (%)						
High school or less	25.70	39.62	27.12	23.39	33.33	<0.001
Junior college	24.74	23.58	22.88	25.95	19.21	
Undergraduate	39.58	25.47	40.96	40.76	35.59	
Graduate	6.00	10.38	5.37	6.16	3.39	
Unknown	3.99	0.94	3.67	3.74	8.47	
Family affluence status (%)						
Low	21.86	19.05	17.51	24.43	11.30	<0.001
Middle	41.09	35.24	47.18	40.48	37.29	
High	33.16	45.71	31.64	31.42	42.94	
Unknown	3.89	0.00	3.67	3.67	8.47	
Sleep Time (%)						
<9 h	58.89	53.85	55.08	59.79	62.15	0.215
≥9 h	41.11	46.15	44.92	40.21	37.85	
Date (%)						
School-day	82.85	81.13	81.07	83.18	84.75	0.667
Weekend	17.15	18.87	18.93	16.82	15.25	

^*^Class 1, “Egg and milk foods”; Class 2, “Grain foods”; Class 3, “Abundant foods”.

### 3.3 Associations between breakfast patterns with executive function

Compared to those who skipped breakfast, participants who consumed breakfast with “Grain foods” had a lower risk of elevated inhibit score (OR: 0.56; 95% CI: 0.31–1.00). The inverse association was attenuated after adjusting for covariates (OR: 0.62; 95% CI: 0.34–1.14). For the same comparison, participants who consumed “Grain foods” and “Abundant foods” had a 57% lower risk (OR: 0.43; 95% CI: 0.25–0.74) and a 64% lower risk (OR: 0.36; 0.17–0.76) of elevated initiate score, respectively. There was a nonsignificant inverse association between “Egg and milk foods” class and an elevated initiate score (OR: 0.56; 95% CI: 0.30–1.03). In contrast, participants who consumed “Egg and milk foods” had a 49% lower risk (OR: 0.51; 95% CI: 0.29–0.88) and a 41% lower risk (0.59; 95% CI: 0.36–0.95) of elevated working memory score. No significant inverse association was observed between “Abundant foods” class and working memory score (OR: 0.64; 95% CI: 0.35–1.19) (Table 3).

**Table 3:** Association between breakfast patterns with executive dysfunction (*n* = 2081).

Executive Dysfunction	Breakfast patterns	Frequency of elevated executive dysfunction	Odds Ratio (95%)	
			Crude Model	Modal^a^
Inhibit	Skipped breakfast	15/106	1.00 (Reference)	1.00 (Reference)
	Class 1*	39/354	0.75 (0.40–1.42)	0.83 (0.43–1.61)
	Class 2*	122/1445	0.56 (0.31–1.00)^#^	0.62 (0.34–1.14)
	Class 3*	16/176	0.61 (0.29–1.28)	0.65 (0.30–1.41)
Shift	Skipped breakfast	14/106	1.00 (Reference)	1.00 (Reference)
	Class 1	36/354	0.74 (0.38–1.44)	0.78 (0.39–1.54)
	Class 2	138/1445	0.69 (0.39–1.25)	0.68 (0.37–1.26)
	Class 3	15/176	0.61 (0.28–1.33)	0.65 (0.29–1.44)
Emotion control	Skipped breakfast	6/106	1.00 (Reference)	1.00 (Reference)
	Class 1	39/354	2.06 (0.85–5.02)	2.09 (0.85–5.12)
	Class 2	130/1445	1.65 (0.71–3.83)	1.57 (0.67–3.69)
	Class 3	23/176	2.51 (0.99–6.37)	2.49 (0.97–6.37)
Initiate	Skipped breakfast	19/106	1.00 (Reference)	1.00 (Reference)
	Class 1	39/354	0.57 (0.31–1.03)	0.56 (0.30–1.03)
	Class 2	137/1445	0.48 (0.28–0.81)^##^	0.43 (0.25–0.74)^##^
	Class 3	13/176	0.37 (0.17–0.77)^##^	0.36 (0.17–0.76)^##^
Working memory	Skipped breakfast	25/106	1.00 (Reference)	1.00 (Reference)
	Class 1	49/354	0.52 (0.30–0.89)^#^	0.51 (0.29–0.88)^#^
	Class 2	235/1445	0.63 (0.39–1.01)	0.59 (0.36–0.95)^#^
	Class 3	29/176	0.64 (0.35–1.16)	0.64 (0.35–1.19)
Plan/organize	Skipped breakfast	16/106	1.00 (Reference)	1.00 (Reference)
	Class 1	62/354	1.19 (0.66–2.17)	1.25 (0.67–2.33)
	Class 2	238/1445	1.11 (0.64–1.92)	1.11 (0.62–1.97)
	Class 3	34/176	1.35 (0.70–2.58)	1.41 (0.72–2.76)
Organization of materials	Skipped breakfast	6/106	1.00 (Reference)	1.00 (Reference)
	Class 1	11/354	0.53 (0.19–1.48)	0.50 (0.18–1.42)
	Class 2	50/1445	0.60 (0.25–1.43)	0.55 (0.22–1.35)
	Class 3	2/176	0.19 (0.04–0.97)^#^	0.18 (0.04–0.94)^#^
Monitor	Skipped breakfast	21/106	1.00 (Reference)	1.00 (Reference)
	Class 1	64/354	0.89 (0.52–1.55)	0.92 (0.52–1.62)
	Class 2	258/1445	0.88 (0.54–1.45)	0.92 (0.55–1.54)
	Class 3	29/176	0.80 (0.43–1.49)	0.83 (0.44–1.58)
BRI	Skipped breakfast	14/106	1.00 (Reference)	1.00 (Reference)
	Class 1	37/354	0.77 (0.40–1.48)	0.84 (0.43–1.67)
	Class 2	133/1445	0.67 (0.37–1.20)	0.68 (0.37–1.27)
	Class 3	19/176	0.80 (0.38–1.66)	0.82 (0.38–1.77)
MI	Skipped breakfast	15/106	1.00 (Reference)	1.00 (Reference)
	Class 1	39/354	0.75 (0.40–1.42)	0.78 (0.40–1.51)
	Class 2	168/1445	0.80 (0.45–1.41)	0.79 (0.43–1.44)
	Class 3	15/176	0.57 (0.26–1.21)	0.60 (0.27–1.31)
GEC	Skipped breakfast	15/106	1.00 (Reference)	1.00 (Reference)
	Class 1	36/354	0.69 (0.36–1.31)	0.75 (0.38–1.46)
	Class 2	132/1445	0.61 (0.34–1.08)	0.63 (0.35–1.16)
	Class 3	16/176	0.61 (0.29–1.28)	0.65 (0.30–1.40)

^*^Class 1, “Egg and milk foods”; Class 2, “Grain foods”; Class 3, “Abundant foods”. #indicates *p* < 0.05. ##indicates *p* < 0.01.

a Adjusted for gender, maternal education, family affluence status, sleep time and school day.

### 3.4 Stratified analyses

In stratified analyses, although the inverse association between breakfast dietary pattern and executive function risk was present across all prespecified groups, including gender, maternal education, and family affluence status (Supplementary Tables S2–S4), a stronger inverse association was observed for girls, participants with lower family affluence status, or those with maternal education below graduate level.

### 3.5 Sensitivity analyses

In the sensitivity analysis, when we excluded those with unknown values, the results were essentially the same (Supplementary Table S5).

## 4 Discussion

In the current study, we identified different breakfast patterns among adolescents aged 12–13 years old in Shanghai, including the “Egg and milk foods” class, “Grain foods” class, and “Abundant foods” class. Adolescents who skipped breakfast tended to have more obvious executive dysfunction than their peers who ate breakfast. Additionally, adolescents who had abundant foods for breakfast were at a lower risk of executive dysfunction.

Breakfast is often described as the most important meal of the day, contributing to 25–30% of total daily energy (31). Brain imaging study indicated that the level of glucose metabolism in the brain is much higher in childhood than in adults (32). The rate of glucose metabolism in the brain remains elevated until 9–10 years of age before it declines to the adult level by late adolescence. A well-balanced diet ensures a continuous supply of glucose to support the high brain metabolism in children (33). Breakfast can help stabilize glucose levels throughout the morning, which improves memory, concentration, and makes students more alert as well (34). In other words, individuals who skip breakfast may experience lower blood sugar levels, leading to decreased cortical excitability and difficulties in concentration (13). Grains are a good source of carbohydrates, which mostly break down into glucose, the brain’s primary energy source (34). We found that over 80% of breakfast-eating participants from the “Grain foods” and “Abundant foods” classes consumed grains such as steamed buns, rice, noodles, wontons, and bread. Additionally, their consumption of grains was positively associated with initiate performance. A Korean intervention study reported that consuming a rice-based breakfast has a positive effect on cognitive function in adolescents who usually skip breakfast (15). A study focused on older adults also found that increased consumption of whole grains was linked to a slower rate of global cognitive decline (35). Further research is needed to determine which specific grain foods have a greater impact on executive function and related mechanisms.

The results showed that 43.67% of participants in the eaten group had eggs for breakfast. Both the “Egg and milk foods” and “Grain foods” classes were associated with superior working memory performance. Eggs are a major source of choline, an essential nutrient that contributes to brain development and function (36). Choline is required to produce acetylcholine, a crucial neurotransmitter for memory, mood, and other brain and nervous system functions (35). A few observational studies have shown a link between higher choline intakes and cognitive performance in adults (37, 38), such as better verbal memory, visual memory, and global cognition. Note that humans cannot produce enough choline to meet daily requirements; it needs to be provided through the diet. Eggs provide one of the highest amounts of choline in any natural foods. A randomized controlled study of children aged 9–14 years indicated that egg yolks resulted in higher short-term learning and memory performance (39). As a primary breakfast food, eggs meet the daily choline requirement and may enhance the executive performance of adolescents throughout the day. Although we found no significant difference between the “Abundant foods” class and the skipped breakfast group on working memory, the odds ratio of the “Abundant foods” class showed a downward trend compared to the skipped breakfast group, which may have been influenced by the sample size. A replication study with a larger sample size should be conducted to validate this finding.

Fluid milk was identified as the primary breakfast item across all three breakfast patterns in the study population. They had better executive performance compared to those who skipped the breakfast, which is consistent with several studies (14, 26, 40). An observational study in Chile reported that adolescents who consumed dairy for breakfast showed higher cognitive performance (14). One study from China found that a high dairy intake was related to better executive function performance compared to low intake among children aged 6–12 (26). An interventional study of overweight and obese adults indicated that a high dairy diet has the potential to improve working memory (40). Milk is rich in a wide range of nutrients, including proteins, fats, and micronutrients such as vitamins B12 and calcium, which are relevant to the brain development and function (41).

In the breakfast-eating group, around 20% of participants had unprocessed red meat (nonruminant) for their breakfast. This food group is typically was found in meat buns, wontons, dumplings, and other foods that contain pork filling. Although red meat is considered a limited food from the perspective of preventing noncommunicable diseases (NCDs) prevention (39), its role in improving child cognitive development cannot be ignored. Previous studies have indicated that higher intakes of unprocessed red meat are associated with better general cognitive ability (40, 42). Red meat contributes a wide range of dietary minerals and vitamins essential for neurocognitive development, such as iron, zinc, and vitamin B12 (43). Iron, for example, plays a major role in brain development by being involved in different enzyme systems (44). Iron deficiency at any stage of life could have adverse effects on neurophysiological function (45). However, the evidence regarding the effects of red meat on the executive function of adolescents was limited (46). It is worth exploring in depth whether red meat might have beneficial effects on specific executive domains for adolescents.

Through stratified analysis, we found that breakfast patterns were significantly more associated with the clinical domains and global executive function of adolescents whose mothers had lower education level. Similar results were presented in the adolescents with lower family affluence status. A cohort study from Australia indicated that a healthy dietary pattern among adolescents was positively associated with higher maternal education level and better family functioning (47). A study conducted in Hong Kong suggested that adolescents from low-income families were more vulnerable to diet-related health issues (48). They received poor food guidance from their family and developed the misconception that “healthy food is expensive” (48). Therefore, adolescents from low social status should be the primary focus of dietary interventions, particularly for breakfast. Improving breakfast patterns can have a greater impact on the executive function of adolescents from low social status compared to their high-status counterparts. It is possible to be a feasible and affordable intervention for low-income families.

The main strength of this study was being the first to identify breakfast patterns and investigate their association with executive function among adolescents China. The findings from this study contribute to an important direction of scientific research, monitoring, and intervention for the local population. There are some limitations in this study. First, this cross-sectional study can only demonstrate the association between breakfast patterns and executive function; it cannot determine a causal relationship. Second, we only investigated one-day breakfast information, which partially reflected breakfast patterns among adolescents in Shanghai. Third, we have not taken into account food intake throughout the day, which could have been a confounding factor between breakfast patterns and executive function performance. The implementation of this study was also affected by the COVID-19 pandemic. As much as possible, we have avoided conducting surveys while student is ill. Even if COVID-19 patients recover, they may experience a reduced appetite, which can impact their breakfast consumption. Finally, since the participants in our study were 12–13 years old, further research is needed to concentrate on adolescents of different age groups and identify changes in breakfast patterns as adolescents grow older.

## 5 Conclusion

In conclusion, we found that a breakfast pattern with abundant foods was associated with good executive function performance among adolescents in China. It is recommended that adolescents should have a variety of foods for breakfast, such as grains, eggs, milk, red meat, and other dietary categories like fruits or nuts. We also demonstrated that breakfast patterns were more strongly linked to executive function in female adolescents and adolescents whose mothers had lower education levels or who came from low-income families. Further research is required to validate our results. If these findings hold true, it is necessary to implement nutritional monitoring and intervene with adolescents who have poor breakfast habits. Breakfast interventions may be a beneficial strategy to reduce the gap in executive function among adolescents from low-income families.

## Data availability statement

The original contributions presented in the study are included in the article/Supplementary material, further inquiries can be directed to the corresponding author.

## Ethics statement

The studies involving humans were approved by Ethics Committee of the Shanghai Municipal Center for Disease Control and Prevention (2022-13). The studies were conducted in accordance with the local legislation and institutional requirements. Written informed consent for participation in this study was provided by the participants’ legal guardians/next of kin.

## Author contributions

XW: Formal analysis, Methodology, Writing – original draft. SQ: Project administration, Visualization, Writing – review & editing. DY: Data curation, Project administration, Writing – review & editing. WQ: Data curation, Writing – review & editing. FZ: Methodology, Supervision, Writing – review & editing. RZ: Data curation, Software, Writing – review & editing. LS: Resources, Writing – review & editing. QY: Investigation, Writing – review & editing. YQ: Investigation, Writing – review & editing. GY: Investigation, Writing – review & editing. CY: Investigation, Writing – review & editing. CL: Conceptualization, Funding acquisition, Supervision, Writing – review & editing.
