# Supplementary material for: Association between breakfast patterns and executive function among adolescents in Shanghai, China
Source: Front Nutr. 2024 May 14;11:1373129. doi: 10.3389/fnut.2024.1373129 (PMC11132186; doi:10.3389/fnut.2024.1373129)
Supplement: Supplementary file 1 [file Table_1.docx]

Supplementary Material

Association between breakfast patterns and executive function among adolescents in Shanghai, China

Xuelai Wang^†^, Shuangxiao Qu^†^, Dongling Yang, Wenjuan Qi, Fengyun Zhang, Rong Zhu, Lijing Sun, Qiong Yan, Yue Qi, Guizhen Yue, Cancan Yin, Chunyan Luo^*^

Division of Child and Adolescent Health, Shanghai Municipal Center for Disease Control and Prevention, Shanghai, China

*** Correspondence:** Chunyan Luo: [luochunyan@scdc.sh.cn](mailto:luochunyan@scdc.sh.cn)

†These authors contributed equally to this work.

Table S1. The BRIEF scores among participants with different breakfast patterns (n=2081).

| Scores | Breakfast patterns (Mean±SD)* | | | |
| --- | --- | --- | --- | --- |
|  | Skipping breakfast | Class 1 | Class 2 | Class 3 |
| Inhibit | 48.26±9.10 | 48.27±8.05 | 47.26±7.49 | 47.21±8.01 |
| Shift | 49.55±9.68 | 49.09±9.41 | 48.74±8.75 | 47.62±9.10 |
| Emotional control | 46.48±8.44 | 46.11±9.64 | 45.81±9.17 | 45.44±10.33 |
| Initiate | 48.72±11.46 | 48.00±9.77 | 47.73±9.18 | 46.30±9.82 |
| Working memory | 50.41±10.05 | 49.82±8.62 | 49.37±8.63 | 48.85±8.86 |
| Plan/organize | 48.49±8.89 | 49.67±8.96 | 49.56±9.08 | 48.72±9.34 |
| Organization of materials | 42.29±8.72 | 43.02±7.73 | 42.79±7.68 | 41.94±7.42 |
| Monitor | 48.03±11.52 | 49.72±9.91 | 49.58±10.16 | 48.40±10.28 |
| BRI | 47.58±9.07 | 47.32±9.12 | 46.67±8.47 | 46.18±9.62 |
| MI | 47.46±10.31 | 48.01±8.94 | 47.71±8.95 | 46.60±9.21 |
| GEC | 47.20±10.27 | 47.54±9.15 | 47.09±8.92 | 46.19±9.67 |

Abbreviations: BRIEF, Behavioral Rating Inventory of Executive Function; SD, Standard Deviation; BRI, behavioral regulation index; MI, metacognition; GEC, global executive composite. * Class 1, ‘Egg and milk foods’; Class 2, ‘Grain foods’; Class 3, ‘Abundant foods’.

Table S2. Association between breakfast patterns with executive dysfunction stratified by gender.

| Executive Dysfunction | Males | |  | Females | |
| --- | --- | --- | --- | --- | --- |
|  | Frequency of EED | Odds Ratio (95%)^a^ |  | Frequency of EED | Odds Ratio (95%)^a^ |
| Elevated inhibit | | | | | |
| Skipped breakfast | 9/63 | 1.00 (Reference) |  | 6/43 | 1.00 (Reference) |
| Class 1* | 16/193 | 0.56 (0.23-1.36) |  | 23/161 | 1.33 (0.46-3.78) |
| Class 2* | 61/682 | 0.61 (0.28-1.32) |  | 61/763 | 0.71 (0.27-1.89) |
| Class 3* | 11/86 | 0.89 (0.34-2.33) |  | 5/90 | 0.45 (0.12-1.68) |
| Elevated shift | | | | | |
| Skipped breakfast | 7/63 | 1.00 (Reference) |  | 7/43 | 1.00 (Reference) |
| Class 1 | 15/193 | 0.65 (0.25-1.72) |  | 21/161 | 0.93 (0.34-2.51) |
| Class 2 | 56/682 | 0.64 (0.27-1.52) |  | 82/763 | 0.71 (0.29-1.75) |
| Class 3 | 6/86 | 0.56 (0.18-1.81) |  | 9/90 | 0.77 (0.25-2.36) |
| Elevated emotion control | | | | | |
| Skipped breakfast | 4/63 | 1.00 (Reference) |  | 2/43 | 1.00 (Reference) |
| Class 1 | 15/193 | 1.29 (0.40-4.10) |  | 24/161 | 3.83 (0.86-17.02) |
| Class 2 | 48/682 | 1.12 (0.38-3.28) |  | 82/763 | 2.60 (0.61-11.02) |
| Class 3 | 10/86 | 2.03 (0.60-6.88) |  | 13/90 | 3.77 (0.80-17.66) |
| Elevated initiate | | | | | |
| Skipped breakfast | 8/63 | 1.00 (Reference) |  | 11/43 | 1.00 (Reference) |
| Class 1 | 10/193 | **0.35 (0.13-0.96)^#^** |  | 29/161 | 0.67 (0.30-1.51) |
| Class 2 | 49/682 | 0.48 (0.21-1.10) |  | 88/763 | **0.38 (0.18-0.79)^#^** |
| Class 3 | 4/86 | 0.35 (0.10-1.23) |  | 9/90 | **0.35 (0.13-0.94)^#^** |
| Elevated working memory | | | | | |
| Skipped breakfast | 12/63 | 1.00 (Reference) |  | 13/43 | 1.00 (Reference) |
| Class 1 | 16/193 | **0.38 (0.16-0.86)^#^** |  | 33/161 | 0.67 (0.30-1.51) |
| Class 2 | 95/682 | 0.67 (0.34-1.34) |  | 140/763 | 0.38 (0.18-0.79) |
| Class 3 | 14/86 | 0.85 (0.36-2.02) |  | 15/90 | 0.35 (0.13-0.94) |
| Elevated plan/organize | | | | | |
| Skipped breakfast | 6/63 | 1.00 (Reference) |  | 10/43 | 1.00 (Reference) |
| Class 1 | 26/193 | 1.54 (0.59-4.01) |  | 36/161 | 1.07 (0.46-2.48) |
| Class 2 | 88/682 | 1.42 (0.58-3.47) |  | 150/763 | 0.91 (0.42-1.97) |
| Class 3 | 17/86 | 2.38 (0.86-6.56) |  | 17/90 | 0.92 (0.36-2.30) |
| Elevated organization of materials | | | | | |
| Skipped breakfast | 1/63 | 1.00 (Reference) |  | 5/43 | 1.00 (Reference) |
| Class 1 | 7/193 | 2.59 (0.30-22.14) |  | 4/161 | **0.16 (0.04-0.66)^#^** |
| Class 2 | 26/682 | 2.72 (0.35-21.07) |  | 24/763 | **0.20 (0.07-0.59)^##^** |
| Class 3 | 2/86 | 1.67 (0.14-19.42) |  | 0/90 | empty |
| Elevated monitor | | | | | |
| Skipped breakfast | 10/63 | 1.00 (Reference) |  | 11/43 | 1.00 (Reference) |
| Class 1 | 34/193 | 1.12 (0.51-2.47) |  | 30/161 | 0.74 (0.32-1.70) |
| Class 2 | 133/682 | 1.25 (0.60-2.58) |  | 125/763 | 0.65 (0.30-1.38) |
| Class 3 | 20/86 | 1.63 (0.69-3.85) |  | 9/90 | **0.35 (0.13-0.97)^#^** |
| Elevated BRI | | | | | |
| Skipped breakfast | 8/63 | 1.00 (Reference) |  | 6/43 | 1.00 (Reference) |
| Class 1 | 13/193 | 0.49 (0.19-1.28) |  | 24/161 | 1.41 (0.50-4.01) |
| Class 2 | 54/682 | 0.55 (0.24-1.24) |  | 79/763 | 0.91 (0.34-2.41) |
| Class 3 | 8/86 | 0.67 (0.23-1.94) |  | 11/90 | 1.10 (0.35-3.44) |
| Elevated MI | | | | | |
| Skipped breakfast | 6/63 | 1.00 (Reference) |  | 9/43 | 1.00 (Reference) |
| Class 1 | 14/193 | 0.74 (0.26-2.06) |  | 25/161 | 0.81 (0.33-1.98) |
| Class 2 | 66/682 | 0.97 (0.39-2.42) |  | 102/763 | 0.68 (0.30-1.54) |
| Class 3 | 7/86 | 0.90 (0.28-2.88) |  | 8/90 | 0.45 (0.16-1.33) |
| Elevated GEC | | | | | |
| Skipped breakfast | 8/63 | 1.00 (Reference) |  | 7/43 | 1.00 (Reference) |
| Class 1 | 14/193 | 0.54 (0.21-1.39) |  | 22/161 | 1.05 (0.39-2.83) |
| Class 2 | 59/682 | 0.63 (0.28-1.43) |  | 73/763 | 0.68 (0.27-1.69) |
| Class 3 | 9/86 | 0.80 (0.29-2.24) |  | 7/90 | 0.56 (0.17-1.80) |

Abbreviations: EED, elevated executive dysfunction. * Class 1, ‘Egg and milk foods’; Class 2, ‘Grain foods’; Class 3, ‘Abundant foods’. ^a^Adjusted for maternal education, family affluence status, sleep time and school day. ^#^ indicates *p* < 0.05, ^##^ indicates *p* < 0.01.

Table S3. Association between breakfast patterns with executive dysfunction stratified by maternal education.

| Executive Dysfunction | Below graduate | |  | Graduate and above | |
| --- | --- | --- | --- | --- | --- |
|  | Frequency of EED | Odds Ratio (95%)^a^ |  | Frequency of EED | Odds Ratio (95%)^a^ |
| Elevated inhibit | | | | | |
| Skipped breakfast | 12/67 | 1.00 (Reference) |  | 3/38 | 1.00 (Reference) |
| Class 1* | 18/177 | 0.55 (0.24-1.25) |  | 21/164 | 1.76 (0.49-6.29) |
| Class 2* | 67/713 | 0.51 (0.25-1.02) |  | 52/678 | 0.99 (0.29-3.34) |
| Class 3* | 5/93 | **0.26 (0.09-0.80)^#^** |  | 10/68 | 2.02 (0.52-7.87) |
| Elevated shift | | | | | |
| Skipped breakfast | 10/67 | 1.00 (Reference) |  | 4/38 | 1.00 (Reference) |
| Class 1 | 14/177 | 0.54 (0.22-1.32) |  | 21/164 | 1.22 (0.39-3.82) |
| Class 2 | 62/713 | 0.57 (0.26-1.21) |  | 72/678 | 0.92 (0.31-2.69) |
| Class 3 | 7/93 | 0.48 (0.17-1.39) |  | 8/68 | 1.09 (0.30-3.90) |
| Elevated emotion control | | | | | |
| Skipped breakfast | 5/67 | 1.00 (Reference) |  | 1/38 | 1.00 (Reference) |
| Class 1 | 17/177 | 1.23 (0.43-3.50) |  | 21/164 | 5.68 (0.73-43.93) |
| Class 2 | 72/713 | 1.23 (0.48-3.19) |  | 57/678 | 3.36 (0.45-25.05) |
| Class 3 | 9/93 | 1.16 (0.37-3.68) |  | 13/68 | **8.78 (1.10-70.29)^#^** |
| Elevated initiate | | | | | |
| Skipped breakfast | 15/67 | 1.00 (Reference) |  | 4/38 | 1.00 (Reference) |
| Class 1 | 20/177 | **0.43 (0.20-0.91)^#^** |  | 18/164 | 1.02 (0.32-3.26) |
| Class 2 | 73/713 | **0.32 (0.17-0.62)^##^** |  | 63/678 | 0.80 (0.27-2.37) |
| Class 3 | 9/93 | **0.36 (0.14-0.89)^#^** |  | 4/68 | 0.50 (0.12-2.17) |
| Elevated working memory | | | | | |
| Skipped breakfast | 21/67 | 1.00 (Reference) |  | 4/38 | 1.00 (Reference) |
| Class 1 | 23/177 | **0.32 (0.16-0.64)^##^** |  | 21/164 | 1.12 (0.36-3.51) |
| Class 2 | 123/713 | **0.41 (0.23-0.73)^##^** |  | 109/678 | 1.48 (0.51-4.29) |
| Class 3 | 14/93 | **0.38 (0.17-0.84)^#^** |  | 14/68 | 2.09 (0.63-6.91) |
| Elevated plan/organize | | | | | |
| Skipped breakfast | 14/67 | 1.00 (Reference) |  | 2/38 | 1.00 (Reference) |
| Class 1 | 36/177 | 1.07 (0.52-2.20) |  | 22/164 | 2.53 (0.56-11.38) |
| Class 2 | 130/713 | 0.83 (0.44-1.60) |  | 104/678 | 2.92 (0.69-12.40) |
| Class 3 | 15/93 | 0.77 (0.33-1.78) |  | 17/68 | **5.71 (1.23-26.52)^#^** |
| Elevated organization of materials | | | | | |
| Skipped breakfast | 4/67 | 1.00 (Reference) |  | 2/38 | 1.00 (Reference) |
| Class 1 | 5/177 | 0.51 (0.13-2.00) |  | 3/164 | 0.30 (0.05-1.91) |
| Class 2 | 24/713 | 0.55 (0.18-1.67) |  | 26/678 | 0.72 (0.16-3.27) |
| Class 3 | 0/93 | Empty |  | 2/68 | 0.49 (0.06-3.73) |
| Elevated monitor | | | | | |
| Skipped breakfast | 18/67 | 1.00 (Reference) |  | 3/38 | 1.00 (Reference) |
| Class 1 | 27/177 | 0.53 (0.26-1.07) |  | 30/164 | 2.51 (0.71-8.79) |
| Class 2 | 139/713 | 0.68 (0.38-1.23) |  | 114/678 | 2.38 (0.71-7.94) |
| Class 3 | 15/93 | 0.58 (0.26-1.28) |  | 11/68 | 2.25 (0.58-8.69) |
| Elevated BRI | | | | | |
| Skipped breakfast | 11/67 | 1.00 (Reference) |  | 3/38 | 1.00 (Reference) |
| Class 1 | 16/177 | 0.54 (0.23-1.28) |  | 20/164 | 1.71 (0.47-6.14) |
| Class 2 | 73/713 | 0.58 (0.28-1.21) |  | 57/678 | 1.07 (0.32-3.62) |
| Class 3 | 7/93 | 0.41 (0.14-1.15) |  | 12/68 | 2.50 (0.65-9.55) |
| Elevated MI | | | | | |
| Skipped breakfast | 13/67 | 1.00 (Reference) |  | 2/38 | 1.00 (Reference) |
| Class 1 | 20/177 | 0.57 (0.26-1.26) |  | 14/164 | 1.47 (0.32-6.87) |
| Class 2 | 88/713 | 0.57 (0.29-1.12) |  | 79/678 | 2.13 (0.50-9.12) |
| Class 3 | 8/93 | 0.43 (0.16-1.13) |  | 7/68 | 1.89 (0.37-9.68) |
| Elevated GEC | | | | | |
| Skipped breakfast | 13/67 | 1.00 (Reference) |  | 2/38 | 1.00 (Reference) |
| Class 1 | 18/177 | 0.49 (0.22-1.10) |  | 14/164 | 1.67 (0.36-7.78) |
| Class 2 | 67/713 | **0.44 (0.22-0.86)^#^** |  | 64/678 | 1.81 (0.42-7.77) |
| Class 3 | 6/93 | **0.29 (0.10-0.83)^#^** |  | 10/68 | 3.03 (0.62-14.73) |

Abbreviations: EED, elevated executive dysfunction. * Class 1, ‘Egg and milk foods’; Class 2, ‘Grain foods’; Class 3, ‘Abundant foods’. ^a^Adjusted for gender, family affluence status, sleep time and school day. ^#^ indicates *p* < 0.05, ^##^ indicates *p* < 0.01.

Table S4. Association between breakfast patterns with executive dysfunction stratified by family affluence status.

| Executive Dysfunction | Low affluence | |  | Middle affluence | |  | High affluence | |
| --- | --- | --- | --- | --- | --- | --- | --- | --- |
|  | Frequency of EED | Odds Ratio (95%)^a^ |  | Frequency of EED | Odds Ratio (95%)^a^ |  | Frequency of EED | Odds Ratio (95%)^a^ |
| Elevated inhibit | | | | | |  |  |  |
| Skipped breakfast | 3/20 | 1.00 (Reference) |  | 6/37 | 1.00 (Reference) |  | 6/48 | 1.00 (Reference) |
| Class 1* | 7/62 | 1.10 (0.20-5.99) |  | 18/167 | **0.61 (0.22-1.68)^#^** |  | 14/112 | 0.90 (0.32-2.56) |
| Class 2* | 31/353 | 0.82 (0.18-3.80) |  | 43/585 | 0.41 (0.16-1.06) |  | 45/454 | 0.70 (0.27-1.77) |
| Class 3* | 0/20 | empty |  | 10/66 | 0.84 (0.28-2.59) |  | 5/75 | 0.45 (0.13-1.57) |
| Elevated shift | | | | | |  |  |  |
| Skipped breakfast | 3/20 | 1.00 (Reference) |  | 4/37 | 1.00 (Reference) |  | 7/48 | 1.00 (Reference) |
| Class 1 | 9/62 | 1.51 (0.29-7.93) |  | 15/167 | 0.74 (0.22-2.44) |  | 11/112 | 0.55 (0.20-1.55) |
| Class 2 | 39/353 | 1.07 (0.23-4.89) |  | 45/585 | 0.56 (0.18-1.70) |  | 50/454 | 0.63 (0.26-1.52) |
| Class 3 | 2/20 | 0.93 (0.11-7.54) |  | 9/66 | 1.16 (0.32-4.21) |  | 4/75 | 0.31 (0.08-1.14) |
| Elevated emotion control | | | | | |  |  |  |
| Skipped breakfast | 1/20 | 1.00 (Reference) |  | 3/37 | 1.00 (Reference) |  | 2/48 | 1.00 (Reference) |
| Class 1 | 11/62 | 4.60 (0.54-39.37) |  | 11/167 | 0.73 (0.19-2.81) |  | 16/112 | 3.30 (0.72-15.14) |
| Class 2 | 32/353 | 1.92 (0.24-15.13) |  | 50/585 | 0.98 (0.28-3.35) |  | 47/454 | 2.19 (0.51-9.46) |
| Class 3 | 2/20 | 2.13 (0.17-26.15) |  | 13/66 | 2.31 (0.60-8.93) |  | 7/75 | 2.17 (0.43-11.05) |
| Elevated initiate | | | | | |  |  |  |
| Skipped breakfast | 7/20 | 1.00 (Reference) |  | 5/37 | 1.00 (Reference) |  | 7/48 | 1.00 (Reference) |
| Class 1 | 11/62 | 0.47 (0.14-1.51) |  | 13/167 | 0.51 (0.17-1.54) |  | 14/112 | 0.60 (0.22-1.68) |
| Class 2 | 43/353 | **0.27 (0.10-0.74)^#^** |  | 50/585 | 0.56 (0.20-1.52) |  | 43/454 | 0.40 (0.16-1.00) |
| Class 3 | 2/20 | 0.22 (0.04-1.26) |  | 6/66 | 0.56 (0.16-2.01) |  | 5/75 | 0.31 (0.09-1.07) |
| Elevated working memory | | | | | |  |  |  |
| Skipped breakfast | 9/20 | 1.00 (Reference) |  | 8/37 | 1.00 (Reference) |  | 8/48 | 1.00 (Reference) |
| Class 1 | 9/62 | **0.27 (0.08-0.89)^#^** |  | 21/167 | 0.50 (0.20-1.25) |  | 14/112 | 0.62 (0.24-1.63) |
| Class 2 | 71/353 | **0.37 (0.14-0.98)^#^** |  | 98/585 | 0.68 (0.30-1.55) |  | 63/454 | 0.71 (0.31-1.62) |
| Class 3 | 2/20 | **0.16 (0.03-0.91)^#^** |  | 16/66 | 1.07 (0.40-2.85) |  | 10/75 | 0.70 (0.25-1.94) |
| Elevated plan/organize | | | | | |  |  |  |
| Skipped breakfast | 5/20 | 1.00 (Reference) |  | 5/37 | 1.00 (Reference) |  | 6/48 | 1.00 (Reference) |
| Class 1 | 10/62 | 0.97 (0.26-3.67) |  | 28/167 | 1.35 (0.48-3.83) |  | 20/112 | 1.19 (0.44-3.27) |
| Class 2 | 73/353 | 1.17 (0.37-3.72) |  | 93/585 | 1.23 (0.46-3.30) |  | 68/454 | 0.99 (0.39-2.47) |
| Class 3 | 4/20 | 1.10 (0.22-5.40) |  | 16/66 | 1.99 (0.65-6.08) |  | 12/75 | 1.09 (0.37-3.19) |
| Elevated organization of materials | | | | | |  |  |  |
| Skipped breakfast | 3/20 | 1.00 (Reference) |  | 1/37 | 1.00 (Reference) |  | 2/48 | 1.00 (Reference) |
| Class 1 | 1/62 | **0.08 (0.01-0.91)^#^** |  | 3/167 | 0.92 (0.09-9.73) |  | 4/112 | 0.61 (0.10-3.56) |
| Class 2 | 16/353 | **0.23 (0.06-0.96)^#^** |  | 15/585 | 1.43 (0.17-11.84) |  | 19/454 | 0.71 (0.15-3.28) |
| Class 3 | 0/20 | empty |  | 1/66 | 0.77 (0.04-13.22) |  | 1/75 | 0.25 (0.02-2.93) |
| Elevated monitor | | | | | |  |  |  |
| Skipped breakfast | 7/20 | 1.00 (Reference) |  | 7/37 | 1.00 (Reference) |  | 7/48 | 1.00 (Reference) |
| Class 1 | 7/62 | 0.31 (0.09-1.10) |  | 30/167 | 0.94 (0.37-2.38) |  | 20/112 | 1.07 (0.41-2.83) |
| Class 2 | 78/353 | 0.68 (0.25-1.90) |  | 105/585 | 0.98 (0.41-2.33) |  | 70/454 | 0.99 (0.41-2.38) |
| Class 3 | 2/20 | 0.27 (0.05-1.59) |  | 13/66 | 1.00 (0.35-2.82) |  | 11/75 | 0.91 (0.32-2.60) |
| Elevated BRI | | | | | |  |  |  |
| Skipped breakfast | 4/20 | 1.00 (Reference) |  | 4/37 | 1.00 (Reference) |  | 6/48 | 1.00 (Reference) |
| Class 1 | 10/62 | 1.13 (0.26-4.89) |  | 10/167 | 0.46 (0.13-1.59) |  | 16/112 | 0.99 (0.36-2.77) |
| Class 2 | 36/353 | 0.60 (0.16-2.27) |  | 42/585 | 0.56 (0.18-1.68) |  | 52/454 | 0.75 (0.30-1.90) |
| Class 3 | 2/20 | 0.60 (0.09-4.25) |  | 10/66 | 1.17 (0.33-4.15) |  | 7/75 | 0.61 (0.19-1.98) |
| Elevated MI | | | | | |  |  |  |
| Skipped breakfast | 7/20 | 1.00 (Reference) |  | 4/37 | 1.00 (Reference) |  | 4/48 | 1.00 (Reference) |
| Class 1 | 7/62 | 0.33 (0.09-1.18) |  | 14/167 | 0.80 (0.24-2.63) |  | 13/112 | 1.07 (0.32-3.55) |
| Class 2 | 53/353 | 0.42 (0.15-1.20) |  | 69/585 | 1.17 (0.40-3.46) |  | 45/454 | 0.88 (0.29-2.65) |
| Class 3 | 2/20 | 0.26 (0.04-1.55) |  | 10/66 | 1.42 (0.41-4.99) |  | 3/75 | 0.36 (0.07-1.71) |
| Elevated GEC | | | | | |  |  |  |
| Skipped breakfast | 6/20 | 1.00 (Reference) |  | 4/37 | 1.00 (Reference) |  | 5/48 | 1.00 (Reference) |
| Class 1 | 6/62 | 0.32 (0.08-1.25) |  | 13/167 | 0.70 (0.21-2.31) |  | 13/112 | 0.93 (0.30-2.82) |
| Class 2 | 39/353 | 0.35 (0.12-1.06) |  | 49/585 | 0.77 (0.26-2.31) |  | 43/454 | 0.74 (0.27-2.03) |
| Class 3 | 2/20 | 0.31 (0.05-1.88) |  | 9/66 | 1.11 (0.31-3.98) |  | 5/75 | 0.54 (0.14-2.00) |

Abbreviations: EED, elevated executive dysfunction. * Class 1, ‘Egg and milk foods’; Class 2, ‘Grain foods’; Class 3, ‘Abundant foods’. ^a^Adjusted for gender, maternal education, sleep time and school day. ^#^ indicates *p* < 0.05, ^##^ indicates *p* < 0.01.

Table S5. Sensitivity analysis of association between breakfast patterns with executive dysfunction (n=1996).

| Executive Dysfunction | Frequency of EED | | Odds Ratio (95%) | |
| --- | --- | --- | --- | --- |
|  |  |  | Crude Model | Modal^a^ |
| Elevated inhibit |  | |  |  |
| Skipped breakfast | 14/103 | | 1.00 (Reference) | 1.00 (Reference) |
| Class 1* | 39/341 | | 0.82 (0.43-1.58) | 0.85 (0.44-1.64) |
| Class 2* | 119/1391 | | 0.59 (0.33-1.08) | 0.62 (0.34-1.13) |
| Class 3* | 15/161 | | 0.65 (0.30-1.42) | 0.64 (0.29-1.39) |
| Elevated shift |  | |  |  |
| Skipped breakfast | 13/103 | | 1.00 (Reference) | 1.00 (Reference) |
| Class 1 | 35/341 | | 0.79 (0.40-1.56) | 0.77 (0.39-1.53) |
| Class 2 | 134/1391 | | 0.74 (0.40-1.36) | 0.67 (0.36-1.24) |
| Class 3 | 15/161 | | 0.71 (0.32-1.56) | 0.69 (0.31-1.54) |
| Elevated emotion control | | |  |  |
| Skipped breakfast | 6/103 | | 1.00 (Reference) | 1.00 (Reference) |
| Class 1 | 38/341 | | 2.03 (0.83-4.94) | 2.06 (0.84-5.05) |
| Class 2 | 129/1391 | | 1.65 (0.71-3.84) | 1.58 (0.68-3.70) |
| Class 3 | 22/161 | | 2.56 (1.00-6.55) | 2.46 (0.96-6.32) |
| Elevated initiate |  | |  |  |
| Skipped breakfast | 19/103 | | 1.00 (Reference) | 1.00 (Reference) |
| Class 1 | 38/341 | | 0.55 (0.30-1.01) | 0.55 (0.30-1.01) |
| Class 2 | 136/1391 | | **0.48 (0.28-0.81)^##^** | **0.43 (0.25-0.74)^##^** |
| Class 3 | 13/161 | | **0.39 (0.18-0.83)^#^** | **0.37 (0.17-0.79)^#^** |
| Elevated working memory | |  | |  |
| Skipped breakfast | 24/103 | | 1.00 (Reference) | 1.00 (Reference) |
| Class 1 | 44/341 | | **0.49 (0.28-0.85)^#^** | **0.46 (0.26-0.81)^##^** |
| Class 2 | 232/1391 | | 0.66 (0.41-1.06) | **0.59 (0.36-0.97)^#^** |
| Class 3 | 28/161 | | 0.69 (0.38-1.28) | 0.66 (0.36-1.24) |
| Elevated plan/organize | | |  |  |
| Skipped breakfast | 15/103 | | 1.00 (Reference) | 1.00 (Reference) |
| Class 1 | 58/341 | | 1.20 (0.65-2.23) | 1.19 (0.63-2.22) |
| Class 2 | 234/1391 | | 1.19 (0.67-2.09) | 1.12 (0.63-1.99) |
| Class 3 | 32/161 | | 1.46 (0.74-2.85) | 1.42 (0.72-2.80) |
| Elevated organization of materials | |  | |  |
| Skipped breakfast | 6/103 | | 1.00 (Reference) | 1.00 (Reference) |
| Class 1 | 8/341 | | 0.39 (0.13-1.15) | 0.37 (0.12-1.12) |
| Class 2 | 50/1391 | | 0.60 (0.25-1.44) | 0.57 (0.23-1.40) |
| Class 3 | 2/161 | | 0.20 (0.04-1.03) | 0.20 (0.04-1.03) |
| Elevated monitor |  | |  |  |
| Skipped breakfast | 20/103 | | 1.00 (Reference) | 1.00 (Reference) |
| Class 1 | 57/341 | | 0.83 (0.47-1.47) | 0.83 (0.47-1.48) |
| Class 2 | 253/1391 | | 0.92 (0.56-1.53) | 0.94 (0.56-1.57) |
| Class 3 | 26/161 | | 0.80 (0.42-1.52) | 0.81 (0.42-1.55) |
| Elevated BRI |  | |  |  |
| Skipped breakfast | 13/103 | | 1.00 (Reference) | 1.00 (Reference) |
| Class 1 | 36/341 | | 0.82 (0.42-1.61) | 0.83 (0.42-1.65) |
| Class 2 | 130/1391 | | 0.71 (0.39-1.31) | 0.68 (0.36-1.26) |
| Class 3 | 19/161 | | 0.93 (0.44-1.97) | 0.87 (0.41-1.86) |
| Elevated MI |  | |  |  |
| Skipped breakfast | 14/103 | | 1.00 (Reference) | 1.00 (Reference) |
| Class 1 | 34/341 | | 0.70 (0.36-1.37) | 0.68 (0.35-1.34) |
| Class 2 | 167/1391 | | 0.87 (0.48-1.56) | 0.81 (0.44-1.47) |
| Class 3 | 15/161 | | 0.65 (0.30-1.42) | 0.64 (0.29-1.40) |
| Elevated GEC |  | |  |  |
| Skipped breakfast | 14/103 | | 1.00 (Reference) | 1.00 (Reference) |
| Class 1 | 32/341 | | 0.66 (0.34-1.29) | 0.67 (0.34-1.32) |
| Class 2 | 131/1391 | | 0.66 (0.37-1.19) | 0.65 (0.35-1.18) |
| Class 3 | 16/161 | | 0.70 (0.33-1.51) | 0.69 (0.32-1.49) |

Abbreviations: EED, elevated executive dysfunction. * Class 1, ‘Egg and milk foods’; Class 2, ‘Grain foods’; Class 3, ‘Abundant foods’. ^a^Adjusted for gender, maternal education, family affluence status, sleep time and school day. ^#^ indicates *p* < 0.05, ^##^ indicates *p* < 0.01.
